# Sacituzumab govitecan response in extensive leptomeningeal carcinomatosis from triple-negative breast cancer: a case report

**DOI:** 10.3389/fonc.2024.1378248

**Published:** 2024-08-12

**Authors:** Jesús Yaringaño, María Roca-Herrera, Simeón Eremiev, Pau Mascaró-Baselga, Pau Benito, Fidel Núñez, Sergi Benavente, Isabel Pimentel

**Affiliations:** ^1^ Medical Oncology Department, Vall d’Hebron Institute of Oncology (VHIO), Hospital Universitari Vall d’Hebron, Barcelona, Spain; ^2^ Radiology Department, Hospital Universitari Vall d’Hebron, Barcelona, Spain; ^3^ Radiation Oncology Department, Vall d’Hebron Institute of Oncology, Hospital Universitari Vall d’Hebron, Barcelona, Spain; ^4^ Breast Cancer and Melanoma Group, Vall d’Hebron Institute of Oncology (VHIO), Hospital Universitari Vall d’Hebron, Barcelona, Spain

**Keywords:** triple-negative breast cancer, leptomeningeal carcinomatosis, antibody-drug conjugate, sacituzumab govitecan, partial response

## Abstract

Sacituzumab govitecan (SG), a Trop-2-directed antibody-drug conjugate (ADC), was the first ADC approved for patients with metastatic triple-negative breast cancer (mTNBC) who had received at least two prior lines of therapy for advanced disease. Although SG has shown promising clinical activity in treating brain metastases in both ASCENT randomized trials and real-world analysis, its utility in leptomeningeal carcinomatosis (LC) remains underexplored. We report the diagnostic and therapeutic process of a patient who develops extensive LC from TNBC treated with SG. She presented a clinical response after the first cycle of SG with a PFS of 6 months. This case report highlights the need for further inquiry into the use of SG in LC.

## 1 Introduction

Triple-negative breast cancer (TNBC) is the most aggressive subtype of breast cancer (BC), morphologically characterized by the lack of expression of the estrogen receptor (ER), progesterone receptor (PR), and human epidermal growth factor receptor 2 (HER2), accounting for 10%–15% of BC. It is associated with a younger age at onset, a higher risk of relapse if a pathological complete response (pCR) is not achieved, and a poor prognosis (1). Its high visceral metastatic potential, mainly to the lung and central nervous system (CNS) is well documented, with a mOS of 10–13 months before the immunotherapy era (2), reaching 23 and 25 months, in the PDL1-positive population only, with chemotherapy in combination with an anti-PD-L1 in the pivotal KEYNOTE-355 and IMpassion130 studies, respectively (3, 4). Within CNS involvement, LC is an infrequent and challenging manifestation of advanced BC (5%), with TNBC being the most frequent subtype to present this complication, accounting for up to 40% of patients who develop CNS metastases (5). The survival rates are dismal, between 4 weeks and 3 months with aggressive multimodal therapy (6).

SG consists of an antibody targeting the human trophoblast cell-surface antigen 2 (Trop-2), an epithelial antigen found in various solid tumors, coupled to SN-38, an active metabolite of irinotecan (7). The initial findings from the IMMU-132–01 trial, a single-arm, open-label, multicenter phase I/II study, which included 512 patients with metastatic epithelial cancer (148 with metastatic TNBC [mTNBC] with a median of three previous lines of therapy), generated its accelerated FDA approval for mTNBC. It demonstrated a response rate of 33%, progression-free survival (PFS) of 5.5 months, and overall survival (OS) of 13.0 months (8, 9).

These findings were consolidated by the ASCENT phase III study. A median PFS (mPFS) of 5.6 months and median OS (mOS) of 12.1 months compared to mPFS of 1.7 months (mPFS HR, 0.41; 95% confidence interval (CI), 0.32 to 0.52; *p* < 0.001) and mOS of 6.7 months (0.48; 95% CI, 0.38 to 0.59; *p* < 0.001) with chemotherapy of physician’s choice (TPC) established SG as the second-line treatment for mTNBC. Moreover, in the subgroup comprising patients with brain metastases, who only represented 12% of the enrollment, SG demonstrated a PFS of 2.8 months versus 1.6 months for TPC (HR, 0.68; 95% CI, 0.38–1.23). There was no improvement in the mOS of 7.0 months with SG versus 7.5 months with TPC (HR, 0.96; 95% CI, 0.55–1.68). Unfortunately, these data are based on a *post-hoc* analysis of a small cohort of patients with brain metastasis only and cannot be applied to LC where, as described before, the prognosis is overshadowing (10–12).

In this case report, we describe a TNBC patient with early CNS relapse previously treated with radiation and systemic chemotherapy presenting with progressive neuropathic pain associated with partial ascending paresis of the lower limbs as a result of LC, treatment strategy, and outcome.

## 2 Case description

A 62-year-old woman with no relevant past medical history was diagnosed with stage IIIC TNBC in February 2021. As part of the GeparDouze phase 3 trial, she received four cycles of triweekly carboplatin AUC5 plus 12 cycles of weekly paclitaxel 80 mg/m^2^, followed by four cycles of epirubicin (E) 90 mg/m^2^ and cyclophosphamide (C) 600 mg/m^2^ ± triweekly atezolizumab 1,200 mg, and underwent radical mastectomy with axillary dissection in September 2021, achieving a pCR. Adjuvant radiation therapy with 48 Gy in 15 fractions was administered, after which she continued on atezolizumab/placebo until February 2022.

In parallel, a germline genomic panel using NGS was performed without any pathogenic or likely pathogenic variants being identified.

**Figure 1:**
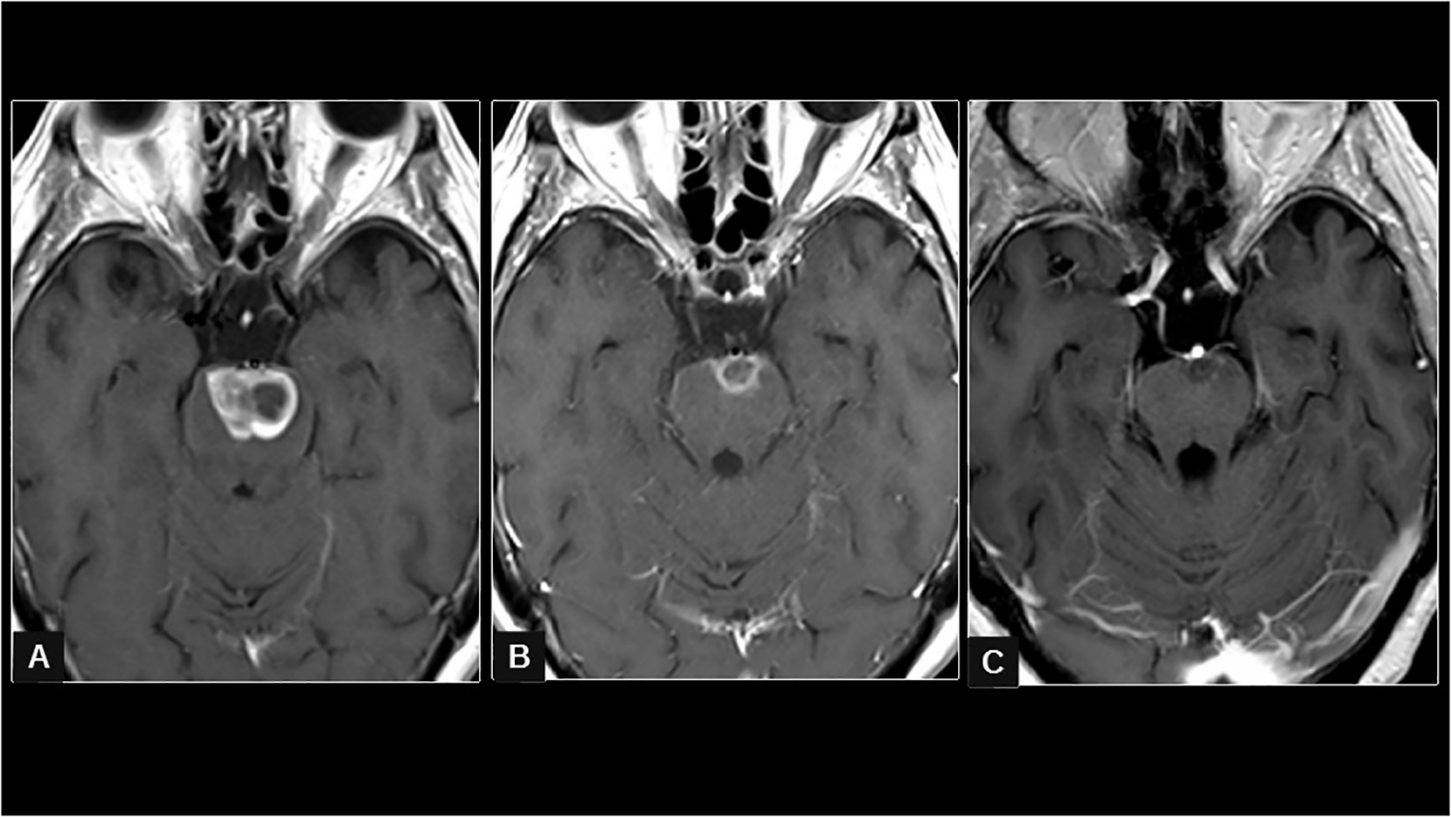
In **(A)** (first brain study from April 2022), the axial T1 sequence after the administration of IV gadolinium shows a contrast-enhancing intra-axial pontine focal lesion with foci of central necrosis and expansion of the brainstem, compatible with M1. In **(B)** (obtained in February 2023, 9 months after the completion of radiotherapy), a reduction in the diameters and degree of uptake of the intra-axial pontine lesion can be seen. The appearance of leptomeningeal uptake is also seen on the pontine surface and cisternal tract of the III nerve bilaterally. In **(C)** (obtained in July 2023, after eight cycles of SG), a notable progressive reduction of the pontine focal lesion and a decrease in leptomeningeal uptake on both the pontine surface and in the III cranial nerve can be seen.

In April 2022, she presented to the emergency room with progressive gait instability and recent dysphagia. A cranial MRI showed a single brainstem lesion and was referred to our center for a second opinion (**Figure 1A**). A Positron emission tomography–computed tomography (PET-CT) was performed and detected two nodules in the mastectomy site (of 7 mm and 4 mm), confirmed with an ultrasound-guided biopsy as TNBC, PD-L1 negative by both Ventana SP263 and CPS score. No other lesions were found on the PET-CT.

Linac-based hypofractionated stereotactic radiotherapy (hSRT) was performed to the metastasis at the level of the pons (28 mm × 21 mm × 29 mm) with 39 Gy in 13 fractions. She received 8 mg/day of dexamethasone during treatment.

After brain radiation, she started first-line treatment for PD-L1-negative metastatic TNBC with weekly paclitaxel 80 mg/m^2^ and triweekly bevacizumab presenting grade 2 (G2) neurotoxicity and switched to capecitabine plus bevacizumab, reaching complete response of the two nodules in the mastectomy site on the PET-CT and partial response by brain MRI that was maintained for 6 months.

In January 2023, she courses in the emergency room after 3 weeks of worsening neuropathic pain in her lower extremities, causing limitations for basic activities associated with recent partial ascending paresis of the lower limbs. She has no fever or any clinical signs of infection. On neurological examination, she was alert, conscious, and oriented, had normal language, preserved naming and repetition, normal eye movements, and motor balance in the lower limbs of the right/left psoas 4−/3+, quadriceps 4−/3+, tibialis anterior 4+/4+, and tibialis posterior 4+/4+. She had preserved arthrokinetic sensitivity, hypopallesthesia in both lower limbs, and absent patellar osteotendinous reflexes.

Upon admission, there were no notable abnormalities in the lab work, and polymerase chain reaction (PCR) was negative for respiratory viruses (COVID, influenza A/B, and respiratory syncytial virus). We performed a first lumbar puncture, and empiric antibiotic treatment was started with ceftriaxone, ampicillin, and acyclovir until microbiology tests resulted negative. The cerebrospinal fluid (CSF) showed low levels of glucose, hyperproteinorrachia, and hypercellularity with 90% lymphocytes (no albuminocytologic dissociation); this is suggestive in this context of leptomeningeal carcinomatosis, since clinically or analytically there were no infectious features.

The electromyogram showed signs of polyradiculoneuritis with demyelinating predominance and a marked distal motor deficit in the lower limbs. The neurophysiologist stated these findings were more suggestive of a dysimmune process or carcinomatous infiltration.

Brain MRI showed an increase in the size of a known right anterior cerebellar pseudonodular enhancement area with perilesional edema, as well as the appearance of small cortico-pial punctate enhancement foci suggestive of metastasis and also showed enhancement of cranial nerves (III, V, and bilateral statoacoustic), which raised the differential diagnosis between a post-radical inflammatory origin versus leptomeningeal involvement. The pontine metastasis maintained an excellent control 9 months after hSRT (11 mm × 8 mm × 9 mm, − 95%) (**Figure 1B**).

After 2 days of admission, the patient deteriorated significantly, becoming unable to walk or even stand up and presented diplopia. Under the clinical and radiological suspicion of cerebral progression with leptomeningeal involvement versus a less likely dysimmune paraneoplastic syndrome with a rapid neurological decline, second-line treatment was started after a shared decision with the patient and her family. The first dose of sacituzumab govitecan (SG) was administered after 5 days of admission.

Later, results from the first lumbar puncture showed no atypical cells in CSF cytology, and autoimmunity markers were negative.

The patient’s lower limb weakness started to improve, along with her pain. We performed a second lumbar puncture and added adenosine deaminase (ADA) quantification in CSF, performing the corresponding cultures as well. ADA was 16 IU/L, cultures were negative, and cytology for a second time did not show malignant cells.

**Figure 2:**
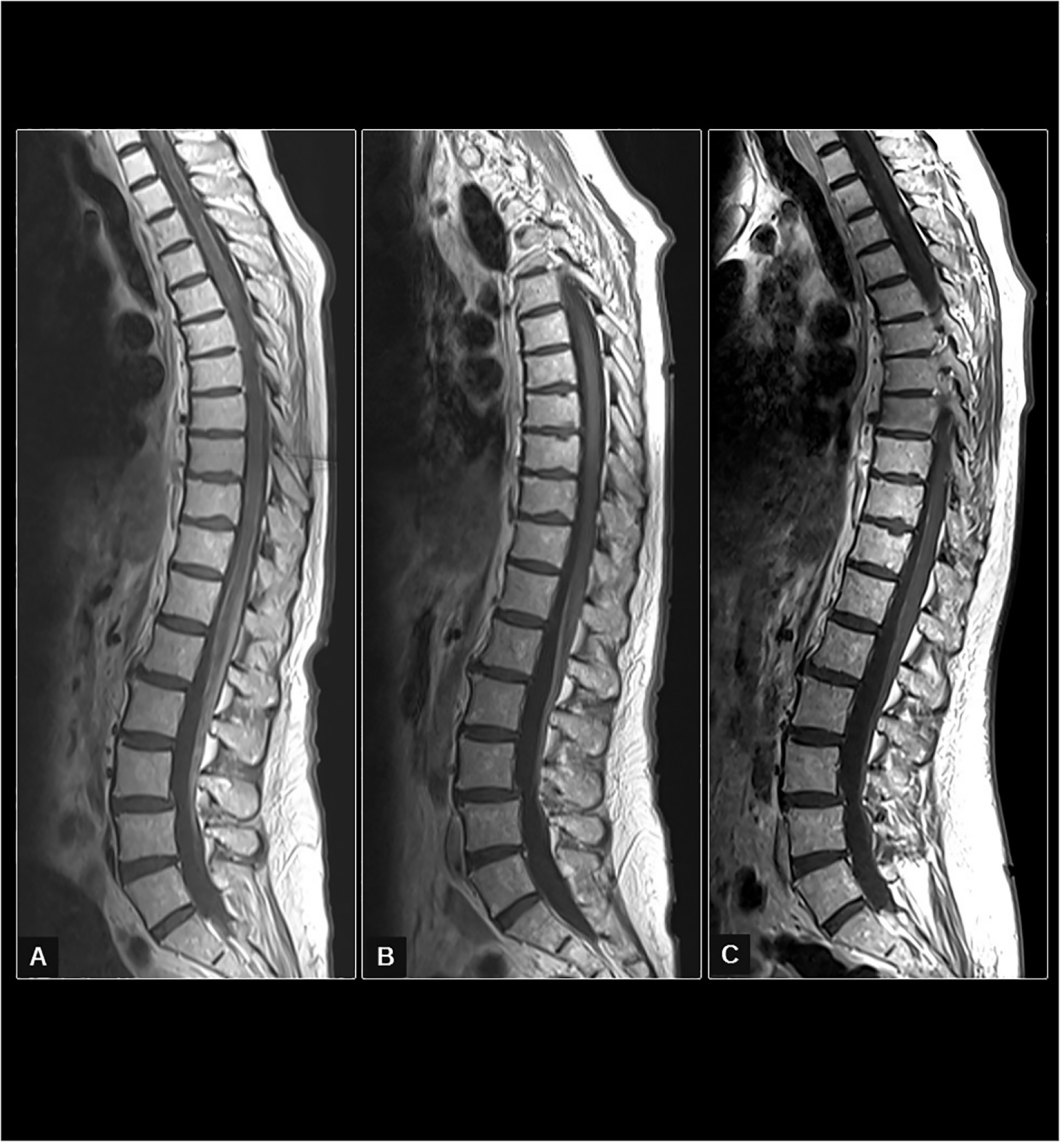
In **(A)** (first spine study in February 2023), the sagittal spine T1 sequence after IV gadolinium administration shows extensive leptomeningeal uptake in the roots of the cauda equina and the surface of the spinal cord, which is compatible with meningeal carcinomatosis. In **(B)** (obtained in April 2023), a moderate decrease in generalized leptomeningeal uptake is seen. In **(C)** (obtained in July 2023), a marked decrease in diffuse leptomeningeal uptake in the cauda equine can be seen. However, a small focal lesion on the surface of the spinal cord at the high dorsal levels is new.

Spinal MRI performed for diagnostic and LC extension showed diffuse and extensive signs of leptomeningeal carcinomatosis, with perimedullary enhancement at the cervical and dorsal spine, as well as conus medullaris and cauda equina (**Figure 2A**). A third lumbar puncture was performed and was again negative.

On day eight of the first cycle of SG, the patient was already able to stand up and started motor rehabilitation, progressively showing better performance in lower limb mobility, and was being discharged from the hospital to a sociosanitary center to continue physiotherapy.

The second cycle of SG was administered as an ambulatory regimen, after which the patient was able to walk longer distances, and the diplopia was completely resolved.

After the third cycle of SG, a CT scan from the thorax and abdomen continued to show no evidence of disease, and the brain and spinal MRI showed partial response with a decrease, in contrast, uptake mostly in the roots of the cauda equina and the surface of the conus medullaris with improvement of the thickening and irregularity seen in the previous study. Moreover, meningeal uptake on the surface of the cord in the rest of its extension, dorsal and cervical, showed a practical resolution (**Figure 2B**).

Subsequently, by cycle four, the patient was fully autonomous and presenting no significant toxicities from the treatment.

She completed eight cycles of SG, and a new brain and spinal MRI was performed, showing a decrease in the diffuse root involvement, although there was a new nodular lesion in the spinal cord at the D3 level, suggesting tumor progression (**Figures 1C**, **2C**). Diplopia and lower limb weakness reappeared a few days after, and treatment was changed to carboplatin AUC5 plus gemcitabine. The patient only received one cycle as her functional status rapidly deteriorated with cephalalgia and confusion, and palliative care was the only option.

She died on September 2023, 7 months after the first dose of SG was administered for extensive and symptomatic LC.

## 3 Discussion

SG has been evaluated in the context of brain metastases, leaving unattended patients with LC, which is increasingly detected (8%), particularly in mTNBC patients guided by the improvement in neuroimaging methods and survival extending (13). Whether to access CNS imaging when the metastatic diagnosis is made is still controversial, and international guidelines recommend only performing it when signs or symptoms of CNS involvement appear, as in the case here reported.

Importantly, LC can be difficult to diagnose by CSF cytology as the first lumbar puncture presents 50%–60% sensitivity, with additional lumbar punctures increasing its sensitivity by 2%–5% per collection (14). In our case, three lumbar punctures were performed and were all negative, but the MRI and electromyography were concordant with the neurologic signs and symptoms of LC. In this context, European Association of Neuro-Oncology (EANO) and European Society for Medical Oncology (ESMO) have proposed a classification of leptomeningeal metastases from solid cancers based on clinical, MRI, and CSF cytology presentation (15). This guideline suggests that patients with type II LC, defined by typical clinical and MRI signs, as the patient here reported, have a better prognosis compared to type I LC, defined by positive CSF cytology (confirmed LC).

Treatment strategies for LC include intrathecal chemotherapy, usually associated with systemic chemotherapy, and radiotherapy, achieving dismal PFS rates from 4 weeks to 3 months (16).

In a real-world data setting, Loirat et al. (17) reported the clinical activity and safety of SG in 103 patients treated through the French Early Access Program from May 2021 to January 2023, including patients with brain metastases, which represented 31.1% of the population. PFS was 4 months (95% CI, 3.5–5.3) and OS was 9.2 months (95% CI, 7.2–NR) with no statistically significant differences in patients with and without brain metastases (12, 17), highlighting the potential for selection bias in randomized controlled trials and the need to complement it with real-world data.

Hanna et al. (18) published another real-world data analysis of 132 patients from 16 UK centers, finding a PFS of 5.2 months and an OS of 8.7 months. A subgroup analysis of 24 patients with CNS disease reported mPFS of 5.1 months; mOS was not reached.

Although there are no data on the LC response to SG, a small window of opportunity trial showed that SG achieves therapeutically relevant intratumoral concentrations of SN-38 in BC brain metastases and recurrent glioblastomas with early intracranial responses. This could be in relation to the molecule’s linker (CL2A) and payload, SN-38 (19).

CL2A is pH-sensitive and allows the detachment of SN-38 at a rate of about 50% per day. Its lower stability allows SN-38 to be released just after SG targets the cell, making it accessible to surrounding tumor cells. The payload, being the active metabolite of irinotecan with a half-maximal inhibitory concentration (IC_50_) in the single-digit nanomolar range for most cell lines, can cross the blood–brain barrier. SG may be able to release SN-38 within the vasculature upon encountering the reduced pH of the tumor microenvironment and crossing the blood–brain barrier, while other ADCs may depend on the presence of disrupted vasculature to reach the tumor antigen and internalization for payload release (19–21).

Unfortunately, patients with LC represent an urgent and unmet clinical need as they are commonly excluded from clinical trials, mainly due to their rapid deterioration and discouraging survival. Sharma et al. (22) conducted a search of the publicly accessible online ClinicalTrials.gov database and found that only eight out of 244 trials allowed enrolment of patients with asymptomatic LC, and all of them were lung cancer trials, highlighting the need for international collaboration.

To the scope of our knowledge, this is the first case reporting SG effectiveness in LC from TNBC. An important and sustained clinical improvement was seen after the first cycle, with a compelling PFS of 6 months, during which the patient fully recovered and had an active life.

This case report emphasizes the need for further research in LC. An important effort should be made to include these patients in future basket trials with emerging treatments that have demonstrated biological plausibility for CNS activity.

## Data Availability

The original contributions presented in the study are included in the article/supplementary material. Further inquiries can be directed to the corresponding author.
